# Efficacy of preemptive intercostal nerve block on recovery in patients undergoing video-assisted thoracic lobectomy

**DOI:** 10.1186/s13019-023-02243-z

**Published:** 2023-04-28

**Authors:** Shaojuan Chen, Zhihua Guo, Xin Wei, Zhenzhu Chen, Na Liu, Weiqiang Yin, Lan Lan

**Affiliations:** 1 Department of Anesthesiology, the First Affiliated Hospital of Guangzhou Medical University, Guangzhou, China; 2 Department of Thoracic Surgery, The First Affiliated Hospital of Guangzhou Medical University, Guangzhou, China; 3 National Clinical Research Center for Respiratory Disease, State Key Laboratory of Respiratory Disease, Guangzhou Institute of Respiratory Health, the First Affiliated Hospital of Guangzhou Medical University, Guangzhou, China

**Keywords:** Video-assisted thoracic surgery, Postoperative pain, Intercostal nerve block, Opioid, Regional anesthesia

## Abstract

**Background:**

Preemptive intercostal nerve block (pre-ICNB) achieves the same analgesic effects as postoperative ICNB (post-ICNB) remains unclear. This study aimed to evaluate the efficacy of preemptive ICNB on perioperative outcomes for patients undergoing video-assisted thoracic surgery (VATS).

**Methods:**

This was a randomized, open-label study (ChiCTR2200055667) from August 1, 2021, to December 30, 2021. Eligible patients scheduled for lobectomy for lung cancer were allocated into the pre-ICNB group and the post-ICNB group. The postoperative pain evaluation, patient rehabilitation, and opioid consumption were observed.

**Results:**

A total of 81 patients were included. When compared with the post-ICNB group, the pre-ICNB group had a lower proportion of hypertension comorbidity (*P* = 0.023), significantly lower total consumption of morphine milligram equivalents (MMEs) (*P* = 0.016), shorter extubation time (*P* = 0.019). The pre-ICNB group has similar Numeric Rating Scales (NRS) scores of dynamic pain in the post-anesthesia care unit (PACU), postoperative 6 h, 12 h, 24 h, and 48 h (*P >* 0.05), and had simialr scores of Bruggrmann Comfort Scale (BCS) in postoperative 6 h, 12 h, 24 and 48 h (*P >* 0.05). The scores of the Mini-mental state examination (MMSE) and Ramsay in the pre-ICNB group were comparable to those in the post-ICNB group, except the scores of MMSE and Ramsay in postoperative 6 h were lower (*P =* 0.048 and *P =* 0.019). The pain evaluation in the 1-month follow-up was comparable with that in the post-ICBN group (*P >* 0.05).

**Conclusions:**

Pre- ICNB is equally efficacious in perioperative pain management as post-ICNB, and pre-ICNB significantly reduces intra-operative opioid consumption, providing faster recovery in PACU.

**Trial registration:**

Registered in the Chinese Clinical Trial Register (ChiCTR2200055667).

**Supplementary Information:**

The online version contains supplementary material available at 10.1186/s13019-023-02243-z.

## Background

Postoperative pain after video-assisted thoracic surgery (VATS) is still considered moderate-severe [1, 2]. However, there is no consensus on the best strategy for its treatment [3, 4]. Local analgesia is strongly recommended for all patients as an integral part of the VATS surgical analgesic protocol [5].

It has been taken for granted that thoracic epidural blockade (TEB), paravertebral blockade (PVB), serratus anterior plane block, and intercostal nerve block (ICNB) are possible techniques for pain management in thoracic surgery [6, 7]. And ICNB is reported to be available for combination with patient-controlled analgesia (PCA) therapy when TEB or PVB has not been performed [8]. However, most local analgesics are usually accepted by surgeons to implement at the end of surgery [5, 9]. Pre-emptive analgesia is a kind of anti-nociceptive treatment. It can prevent altering the afferent process, which exacerbates postoperative pain. At the beginning of the 20th century, Crile [10] put forward the concept of preemptive analgesia based on clinical observations, which can prevent intraoperative nociception and the formation of painful scars caused by physiologic changes resulting in central sensitization and amplification of pain signals [3, 4]. Therefore, preemptive local anesthesia may be more effective than postoperative local anesthesia administration at preventing postoperative pain. Few studies compare the effect of pre-ICNB and post-ICNB on VATS, and some trials also draw contradictory results that pre-emptive analgesia may be effective or ineffective on post-thoracotomy pain syndrome (PTPS) [11–14].

Hereupon, we analyzed the efficacy of preemptive ICNB combined with intravenous PCA as multimodal analgesia for patients undergoing thoracoscopic lobectomy on the postoperative pain score, rehabilitation, intraoperative amount of opioid consumption, and the early incidence of postoperative chronic pain.

## Methods

This study was a single-center prospective randomized controlled trial, which was conducted following the principles outlined in the Declaration of Helsinki.

### Patients

Patients aged 18 ~ 70 years underwent elective anatomic lobectomy by VATS for lung cancer from Aug 1, 2021 to Dec 30, 2021. Exclusion criteria included previous thoracic surgery, emergency or urgent surgery, unilateral multiple lung masses, preoperative radiotherapy or chemotherapy, alcohol abuse, chronic narcotic use, fibromyalgia, preoperative use of analgesics or sedatives, hepatic dysfunction, renal failure, moderate and severe chronic obstructive pulmonary disease (forced expiratory volume in one second in predicted < 80%), cardiac dysfunction (ejection fraction < 50%), conversion to open thoracotomy or serious surgical complications (such as massive bleeding, respiratory failure, etc.).

### Grouping, randomization, and blinding

Patients were randomly allocated into two groups according to the random ID: the preoperative intercostal nerve block group (Group pre-ICNB): intercostal nerve block was implemented before lung resection; and the postoperative intercostal nerve block group (Group post-ICNB): intercostal nerve block was implemented at the end of the operation. The patients, the researchers at postoperative follow-up, and the statisticians were blinded to the randomization.

### Anesthesia management

All patients received general anesthesia with double-lumen endobronchial intubation, which anesthesia induction with propofol, sufentanil, and cisatracurium, and maintained with total intravenous anesthesia of propofol, dexmedetomidine, remifentanil, and cisatracurium. The anesthetics were adjusted to maintain the value of a bispectral index (BIS) at 50 ± 10; Heart rate and systolic blood pressure were controlled within 20% of the baseline value. Atropine or phenylephrine was administered if the heart rate dropped below 50 bpm or the systolic blood pressure decreased below 90mmHg.

### Surgical procedure

As previously described, the procedures of VATS were performed with 2 ports [15]. That means placing a 10 mm camera port on the sixth intercostal space at the mid-axillary line for using a 30-degree angled camera, and a 3 cm access incision on the fourth intercostal space at the anterior axillary line for the surgical approach, without rib resection or rib spreading. A soft incision protector was placed maximize the intercostal spaces to safeguard the skin, subcutaneous tissue, rib, and pleura. VATS lobectomy + lymph node dissection (including lymph nodes of more than 3 stations and N2 for a total of 6 stations) was performed. At the end of the operation, a thoracic drain was inserted into the camera incision. All patients were sent to the post-anesthesia care unit (PACU) and then transferred to the ward when their vital signs were stable.

When there was no air leakage and the drainage flow was less than 150 ml/ day, the drainage tube was removed. Then patients performed a chest X-ray examination and determined whether they can discharge according to the examination results.

### Pain management strategy

All the pre-emptive block procedures were done by surgeons. In the pre-ICNB group, the intercostal block scheme was that 2% lidocaine 3ml was injected into the skin incision before the operation, and 2ml of 0.75% ropivacaine + 2% lidocaine was injected in each intercostal space under visual control by 30° degree camera immediately after entering the chest cavity, with the local eminence of intercostal space, covering the intercostal nerves III–IX. In the post-ICNB group, the block of skin incision and intercostal space were done at the end of the lung resection. The drug dosage and procedures were the same as in the pre-ICNB group.

In the recovery room, all patients received a PCA pump with morphine 1 mg/kg + tropisetron 10 mg with a total of 100 mL 0.9% normal saline, a background dose of 0.5mL, a self-administered bolus dose of 3mL, locking time of 20 min, and limited record 10ml/ h.

### Pain evaluation

In the PACU and the surgical ward after the operation, another blinded researcher conducted pain assessments by using the Numeric Rating Scales (NRS) score of dynamic pain, which was at the guidance by the cutpoints as suggested by Serlin [16], 0 indicating no pain, 1–3 meaning mild pain, 4–6 meaning moderate pain, and 7–10 meaning severe pain. 100 mg of tramadol (Toradol; Pfizer Pharmaceutical, New York, NY) was given by intramuscular injection if the patients’ NRS > 6 during activity, and the pain was reassessed after 1 h. If the NRS score was also greater than 4, tramadol 100 mg was given again. The NRS score mainly assessed incisional pain. If the remedial analgesia, including tramadol or other stronger analgesics, were used, the times of remedial therapy would be recorded. The Bruggrmann comfort scale (BCS) was used to evaluate postoperative pain. It is divided into 5 levels, such as persistent pain, severe pain during deep breathing and coughing, slight pain during deep breathing and coughing, and no pain during deep breathing and coughing. Mini-mental state examination (MMSE) acted as a method for cognitive impairment, and when the score was less than 24 was regarded as dementia. Ramsay scale was used to assess the degree of sedation, it divided into different levels of fidgety, quiet and cooperative, and drowsiness, while a quick response to instruction. The total dosage of opioid consumption was converted to intravenous morphine milligram equivalents (MMEs).

### Study end points

All the perioperative data of patients were included, including the preoperative characteristics, such as body mass index (BMI), revised cardiac risk index (RCRI), values of pulmonary function tests, etc., and the use of intraoperative drugs, the recovery in the PACU, in the ward, and 1-month follow-up. The duration of anesthesia referred to the time from anesthesia induction to the patient being sent out of the operating room, and the duration of surgery referred to the time from skin incision to skin closure and coverage on the incision.

The primary endpoint of the study was the pain and sedation score, which was evaluated as the score of NRS of dynamic pain and conscious time in PACU, the scores of NRS of dynamic pain, BCS, MMSE, and Ramsay on postoperative 6 h, 12 h, 24 h, and 48 h, and the characteristics in postoperative recovery. Secondary endpoints included the total amount of opioid consumption and remedial analgesia in the ward and the pain evaluation of follow-up by telephone 1 month after the operation.

### Statistical analysis

#### Sample size

The sample size of this study was calculated by PASS 11 program. Referring to the previous research results [17], the postoperative pain score was 1.9 ± 0.6 vs. 2.7 ± 0.5 in the internal intercostal nerve block group and the controlled group without block, it would need to study 12 experimental subjects and 12 control subjects to be able to reject the null hypothesis that the population means of the experimental and control groups are equal with probability (power) 0.9. The Type I error probability associated with this test of the null hypothesis is 0.05. Considering the 10% withdrawal rate of a potential patient, it was decided to include at least 13 patients in each group.

### Data analysis

The statistical analysis was performed by SPSS. 26 (IBM Corp., Armonk, NY, USA). A Kolmogorov–Smirnov test was used for analyzing distribution. When the data was normally distributed, it was presented as mean ± standard deviation, and Independent-Samples T-Test was used to compare the differences in outcome parameters. Continuous data with non-parametric dispersion was presented as median and interquartile ranges and was analyzed by the Mann-Whitney U-test to assess the differences between groups. Dichotomous variables were presented as percentages of the total number (%) and were evaluated using the chi-squared test. A *P*-value < 0.05 was regarded as statistical significance.

## Results

**Fig. 1:**
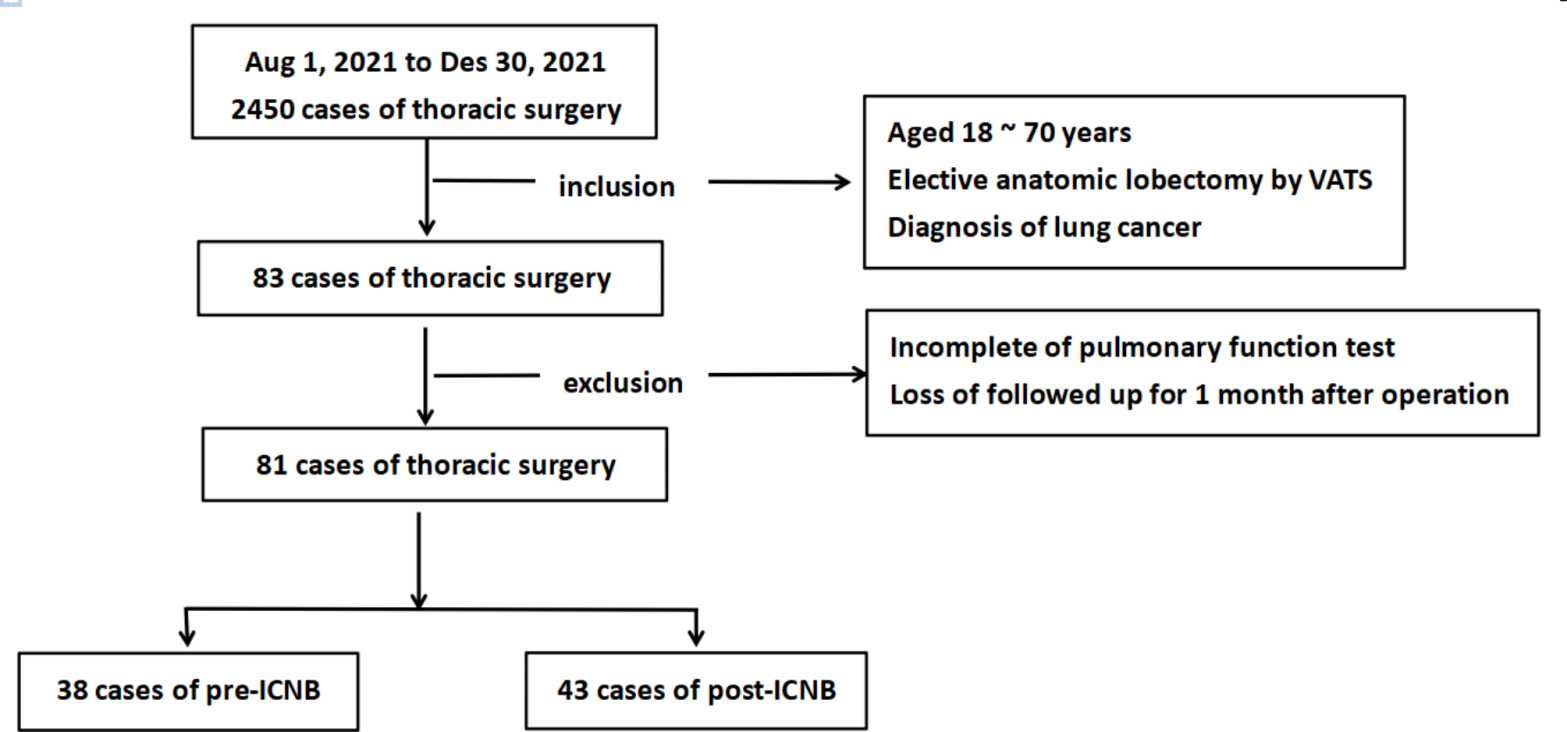
The flowchart of the study

A total of 81 patients were analyzed, with 38 in the pre-ICNB group and 43 in the post-ICNB group. Two patients in the pre-ICNB group were excluded from the study due to incomplete clinical data of pulmonary function tests and lose of follow-up for 1 month after the operation. (Fig. 1)

There was no difference in age, BMI, gender,RCRI, left ventricular ejection fraction, values of pulmonary function tests, surgical location, surgical site, and tumor size, except for the lower proportion comorbidity of hypertension in the pre-ICNB group (*P* = 0.023). (Table 1)

**Table 1:** Preoperative characteristics of patients

Variable	Pre-ICNB group (n = 38)	Post-ICNB group (n = 43)	*P* value
Median Age (y)	58.08 ± 9.02	60.16 ± 8.50	0.288
BMI (kg/m^2^)	22.99 ± 3.16	23.89 ± 3.0	0.195
Gender (n, %)			0.494
Male	17 (44.7%)	16 (37.2%)	
Female	21 (55.3%)	27 (62.8%)	
Comorbidity (n, %)			
Hypertension	1(2.6%)	8 (18.6%)	0.023
Diabetes	1 (2.6%)	4 (9.3%)	0.216
Neurological disease	1 (2.6%)	0	0.287
Pulmonary disease	1 (2.6%)	1(2.3%)	0.930
ASA score (n, %)			0.051
I	34 (87%)	31 (72.1%)	
II	4 (13%)	12 (27.9%)	
Revised cardiac risk index (n, %)			0.287
1 point	37 (97.4%)	43 (100%)	
2 points	1 (2.6%)	0	
LVEF value%	71.02 ± 6.80	69.95 ± 5.38	0.434
Pulmonary function tests (%)			
FVC % predicted	101.28 ± 13.80	102.83 ± 23.37	0.715
FEV_1_% predicted	93.42 ± 15.38	99.04 ± 18.59	0.149
Surgical location (n, %)			0.195
Upper lobe	19 (50%)	27 (62.8%)	
Middle lobe	5 (13.2%)	6 (14.0%)	
Lower lobe	14 (36.8%)	10 (23.3%)	
Surgical site (n, %)			0.176
Left side	18 (47.4%)	14 (32.6%)	
Right side	20 (52.6%)	29 (67.4%)	
Tumor size (mm)	22.18 ± 7.40	20.21 ± 10.65	0.342

BMI, Body mass index; ASA, American Society of Anesthesiologists; LVEF, Left ventricular ejection fraction; RCRI, Revised cardiac risk index; FVC% predicted, the Forced vital capacity in percent of predicted; FEV_1_% predicted, the Forced expiratory volume in the first second in percent of predicted.

During the operation, the dosage of propofol (628 vs. 712 ugs), dexmedetomidine (56.29 vs. 58.49 ugs), midazolam (2.86 vs. 2.84 mg), and cisatracurium (19.95 vs. 23.79 mg) had no difference between the pre-ICNB group and post-ICNB group (Supple Table 1), while the dosage of MMEs (83.8 vs. 101.67 mg) was less in the pre-ICNB group (*P* = 0.016). The duration of surgery and anesthesia had no difference between groups (101.84 vs. 109.49 min, *P* = 0.284 and 177.89 vs. 182.37 min, *P* = 0.600) (Table 2).

**Table 2:** The comparison of MMEs and postoperative recovery

Variable	Pre-ICNB group (n = 38)	Post-ICNB group (n = 43)	*P* value
MMEs (mg)	83.8 ± 30.73	101.67 ± 36.70	0.016
Duration of surgery (min)	101.84 ± 25.37	109.49 ± 36.55	0.284
Duration of anesthesia (time)	177.89 ± 35.39	182.37 ± 40.51	0.600
Extubation time (min)^b^	21 (15, 31.25)	28 (20, 35)	0.019
Concious time (min)^b^	10 (5, 13.25)	10 (5, 15)	0.391
Duration of PACU (min)	77.97 ± 21.61	80.26 ± 29.30	0.694
Remedial analgesia in the wards (n, %)			0.623
No	13 (34.2%)	17 (39.5%)	
Yes	25 (65.8%)	26 (60.5%)	
Frequency of pressing PCA^b^	4 (2, 7)	4 (2, 6)	0.614
Chest-tube duration (h)	41.58 ± 12.35	42.81 ± 9.30	0.617
Time to flatus (h)	17.61 ± 8.49	15.86 ± 8.25	0.351
Drinking time (h)	11.89 ± 4.87	10.79 ± 5.35	0.337
Postoperative ambulation time (h)	21.74 ± 7.42	23.16 ± 7.96	0.409
Day of discharge (h)	56.68 ± 12.0	60.33 ± 12.39	0.184

MMEs, Morphine milligram equivalents; PACU, Post-anesthesia care unit; PCA, Patient-controlled analgesia

^b^ Median (interquartile range)

In the postoperative recovery, the patient’s conscious time, the duration of PACU, the remedial analgesia, the frequency of pressing the PCA pump, chest-tube duration, time to flatus, drinking time, postoperative ambulation time, and the day of discharge had no difference between two groups (*P* > 0.05), except the extubation time was less in the pre-ICNB group when compared with the post-ICNB group (21 vs. 28 min, *P* = 0.019). (Table 2)

In the ward, all patients had no skin itching, urine retention, and delirium. The incidence of postoperative nausea or vomiting was similar between the two groups (*P* > 0.05). (Table 3)

**Table 3:** The incidence of nause or vomiting

Variable	Pre-ICNB group (n = 38)	Post-ICNB group (n = 43)	*P* value
Post-op 6 h (n, %)			0.800
No	31 (81.6%)	36 (83.7%)	
Yes	7 (18.4%)	7 (16.3%)	
Post-op 12 h (n, %)			0.800
No	31 (81.6%)	36 (83.7%)	
Yes	7 (18.4%)	7 (16.3%)	
Post-op 24 h (n, %)			0.491
No	36 (94.7%)	39 (90.7%)	
Yes	2 (5.3%)	4 (9.3%)	
Post-op 48 h (n, %)			0.099
No	38 (100%)	40 (93.0%)	
Yes	0	3 (7.0%)	

Post-op, Post-operative

**Fig. 2:**
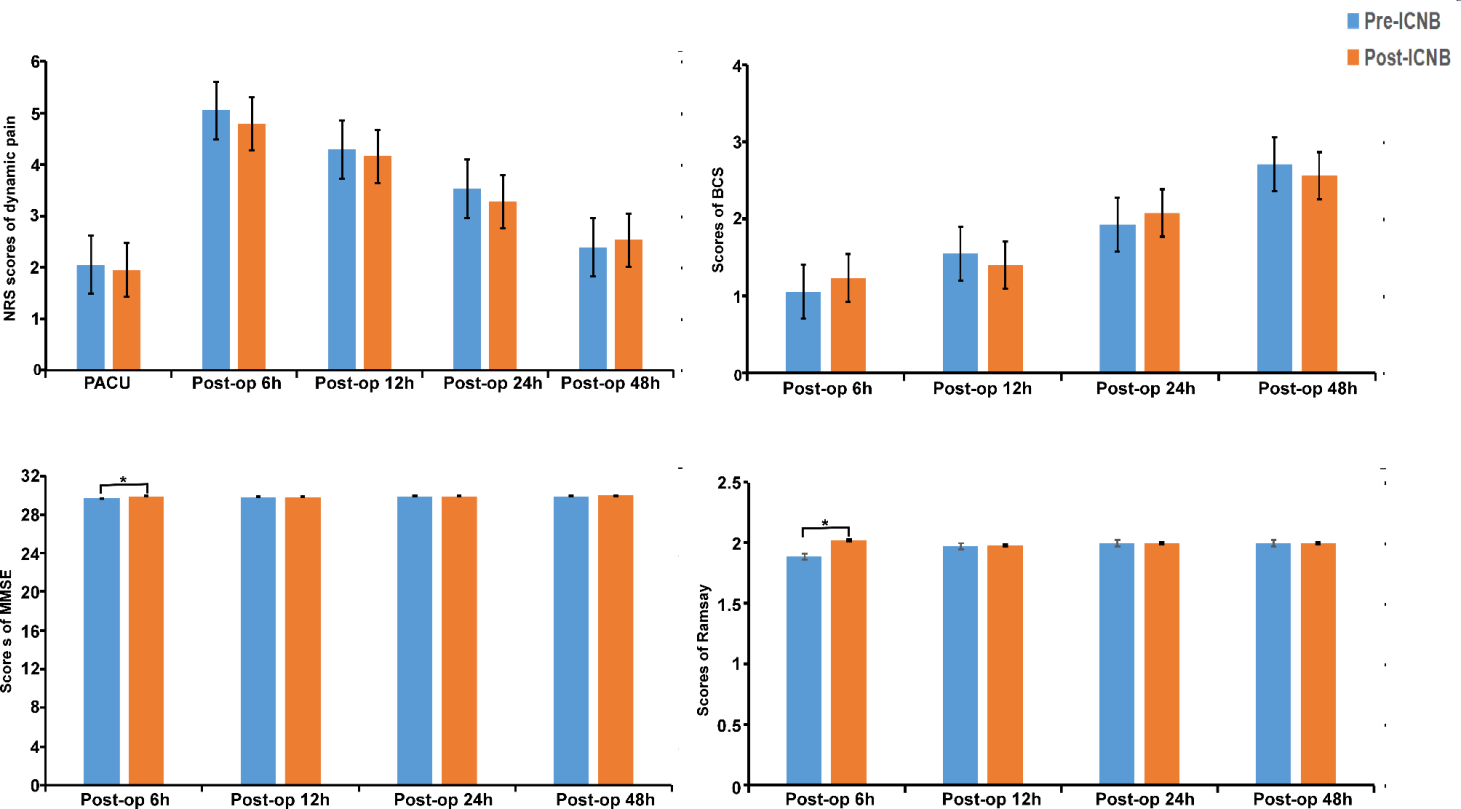
The scores of NRS of dynamic pain, BCS, MMSE, and Ramsay in postoperative evaluation. ^*^P < 0.05 presented the comparison between the pre-ICNB and the post-ICNB group. When compared with scores in the post-ICNB group, the pre-ICNB group had a lower score of MMSE (*P =* 0.048) and a lower incidence of fidgety in Ramsay score (*P =* 0.019) in postoperative 6 h. NRS, Numeric rating scales; BCS, Bruggrmann comfort scale; MMSE, Mini-mental state examination

When compared with the post-ICNB group, the pre-ICNB group had similar NRS scores of dynamic pain in the PACU, postoperative 6 h, 12 h, 24 h, and 48 h (*P >* 0.05), and had comparable scores of BCS in postoperative 6 h, 12 h, 24 and 48 h (*P >* 0.05). The scores of MMSE and Ramsay in the pre-ICNB group were comparable to those in the post-ICNB group, except for the lower score of MMSE (*P =* 0.048) and lower incidence of fidgety in Ramsay score (*P =* 0.019) in postoperative 6 h. (Fig. 2)

In 1 month follow-up after the operation, there was no difference in the type of pain, the incidence of oral analgesics, the NRS of dynamic pain, and the impact of pain on daily life between the pre-ICNB group and the post-ICNB group (*P >* 0.05). (Table 4)

**Table 4:** The pain evaluation of 1 m follow-up after operation

Variable	Pre-ICNB group (n = 38)	Post-ICNB group (n = 43)	*P* value
Type of pain (n, %) None Dull pain Traction pain Pain at rest Pressing pain	15 (39%) 2 (5%) 6 (17%) 15 (39%) 0	22 (51%) 4 (9%) 5 (12%) 10 (23%) 2 (5%)	0.278
Oral analgesics (n, %) No Yes	36(95%) 2 (5%)	41(95%) 2 (5%)	0.900
NRS of dynamic pain (n, %) ≤ 3 4–6 ≥ 7	20 (53%) 17 (45%) 1 (2%)	26 (61%) 16 (37%) 1 (2%)	0.488
Impact of pain on daily life (n, %) None Slight Affect sleep	35 (92%) 2 (5%) 1 (3%)	40 (93%) 3 (7%) 0	0.851

## Discussion

The present study compared the efficacy of preemptive ICNB in patients undergoing VATS lobectomy with post-operative ICNB. The method of pre-ICNB decreased the dosage of MMEs, resulting in a faster extubation in PACU. The recovery in the ward of pre-ICNB was comparable to post-ICNB, it had comparable postoperative pain scores of NRS of dynamic pain and BCS. The evaluation scores of sedation in the pre-ICNB group were also similar to those in the post-ICNB group, except for the lower score of MMSE and lower incidence of fidgety in postoperative 6 h. In 1 month follow-up, there was also no early indication of postoperative chronic pain happened.

Compared with thoracotomy, VATS leads to fewer complications, less anesthetic use, and shorter hospital stay [18], but it may still have a significant correlation with postoperative pain. Sufficient perioperative analgesia can decrease the rate of postoperative morbidity and mortality, and reduce the incidence of chronic pain after thoracic surgery [19]. Regional analgesic techniques are considered an important part of multimodal analgesia. They can alleviate pain more effectively than systemic opioids alone and can decrease opioid consumption, which contribute to reducing opioid-related adverse effects [3, 4].

In this observation, pre-ICNB resulted in rapid rehabilitation with faster extubation in PACU, due to much less use of intraoperative analgesic drugs. Pre-ICNB implemented before operation can decrease the dosage of intraoperative opioid drugs by 22% in this study, which resulted in the same result as *Ponholzer’s* [20]. It is no doubt that the reduction of intraoperative analgesics is more helpful to stabilize hemodynamics in severe or elderly patients [21], mitigating the risk of postoperative pneumonia and atelectasis in thoracic patients, and decreasing the incidence of needing intensive care unit [22, 23]. In addition, in the current wave of opioid prevalence, it is particularly important to pay attention to the side effects of opioids in postoperative pain management and the possibilities of reducing their use [24, 25]. Pre-ICNB might not only affect patient satisfaction but also combat the increasing budget pressure of hospital medical service, which is a necessary state of rehabilitation. The pre-ICNB method had comparable the time of anesthesia and surgery, and the recovery in the ward with post-ICNB. As such, pre-ICNB may be an attractive choice for regional analgesic intervention for post-VATS pain.

Preemptive ICNB is proven to provide equivalent pain relief to TEB during operation [26]. However, the ICNB is often accepted by surgeons to block after the operation considering the pharmacodynamics of local anesthetics [3, 20]. Lidocaine and ropivacaine are commonly applied to intercostal nerve block owing to their simplicity and low cost. The duration of the block is usually 6 to 8 h, which is relatively short. The preoperative block needs to consider the duration of analgesia. While interesting, early implementation of pre-ICNB may offer the same pain relief as the post-ICNB method in this study. It led to comparable NRS scores of dynamic pain in PACU, postoperative 6 to 48 h, similar BCS scores in postoperative 6 to 48 h, also a similar requirement for pressing the PCA pump and remedial therapy in the ward when compared with those in the post-ICNB. This proved that pre-ICNB can provide the same analgesic effect as post-ICNB, and also meet the analgesic requirements of 48 h after the operation. It may be considered a suitable alternative to post-ICNB. Thoracic surgeons should consider changing their current habits to accept the preemptive ICNB for maximum benefits.

TEB usually acquires good management of pain control [6], but it is difficult to perform, time-consuming, and with potentially serious side effects [27]. Therefore, the application ofepidural analgesia in VATS is still a controversial issue [28], and may not be necessary for minimally invasive thoracoscopic surgery [28, 29]. Other alternatives, including PVB, serratus anterior plane block, and ICNB, are also possible [7]. Though the surgeon can place the PVB under VATS, publications of such techniques are uncommon. The intercostal catheter may be the latest and most effective postoperative analgesia. However, it is still worried about catheter infections, and poor analgesia caused by catheter displacement, and it needs certain practice to successfully implement this method [20]. ICNB is convenient for thoracic surgeons to perform without a learning curve and no need for ultrasound. it takes approximately less than 5 min to perform under direct vision, and does not result in epidural anesthesia or paravertebral analgesia related to a hematoma, nerve damage, or pneumothorax. It has been proved that ICNB is an alternative option to more invasive blocks such as TEB or PVB [30]. ICNB may be more suitable for ultimate minimally invasive sole-port thoracoscopic surgery than other pain relief methods with obvious invasive and complicated procedures.

The scope of our block was wider than others [31], and the dosage blocked for each intercostal space was less. First, in addition to the intercostal nerve block, it was also performed local block in manipulating and observing ports before skin incision to enhance the analgesic effect. Second, there is less space in each coastal space. If more volume is injected, most drugs will leak out and decrease the blocking effect. Therefore, it was usually only injected 2-3ml in each intercostal space [32]. Finally, although the operation ports are at the 6th and 4th intercostal sites, there is a cross-link between the upper and lower intercostal nerves and the visceral nerves of the lung have a wide range of innervation. Therefore, blocking the range of 2–3 intercostal nerves higher or lower than the operation ports would strengthen the analgesic effect. The drug composition of the intercostal block was also different from other’s reports [31]. The intercostal block of lidocaine combined with ropivacaine is conducive to the rapid onset of pre-emptive analgesia, avoiding the need to increase intravenous opioids at the beginning of the operation, and maintaining hemodynamics more stability.

Chronic postoperative pain is a common and serious complication after thoracic surgery. Its incidence is variable, 9–80% after thoracotomy, and 5–33% after VATS [33]. Regional anesthesia, as a method of preemptive analgesia, can decrease the rate of postoperative chronic pain [34]. Two of the previous RCTs [11, 12] find the pre-emptive effect of TEA to reduce PTPS. However, two multimodal therapy of prospective randomized trials, including intercostal nerve blocks [13, 14] do not find that pre-emptive analgesia had an advantageous effect on PTPS. In the 1-month follow-up in this study, the pre-ICNB had no difference in the type and degree of pain, the incidence of oral analgesics, and the impact of pain on daily life when compared with the post-ICNB. This result was similar to that of Ozyalcin [35], which has also been chosen to evaluate PTPS at 1 month. Although chronic pain after thoracotomy is mostly defined as the pain that recurs or persists at least 2 months following the surgical procedure [36], a one-month follow-up could distinguish the chronic pain earlier and expel the influence factors, such as the patient’s amnesia, anxiety, or depression.

### Limitations

This study has several limitations. First, it mainly evaluated acute postoperative pain during hospitalization, and only 1 month of follow-up was carried out. It takes at least 3 weeks for chronic pain to occur. Therefore, it may have different results to prolong the postoperative follow-up time on chronic pain. Second, although propensity score matching was not performed, this was a randomized-blinded study to reduce the potential bias among groups. The researchers of follow-up and the data analyst did not know about the grouping. Third, the number of patients in each group was small, but the sample power was calculated according to the previous study to minimize the impact on the result. Fourth, it was a lack of evaluation of the neuropathic complications after thoracoscopy (i.e. paraesthesia, postoperative pulmonary function), and these evaluation items need to be added in future observation. In this study, only the conscious time, and the score of MMSE and Ramsay were evaluated.

## Conclusions

Preemptive ICNB has equivalent pain relief, and similar analgesic-related adverse effects as post-ICNB, and it is a suitable method in combination with PCA as multimodal analgesia to reduce opioid consumption and improve the faster extubation in PACU when undergoing VATS. Surgeons should consider changing their habits to implement this simple and safe preemptive ICNB. A preemptive nerve block may bring better benefits to patients.

## Electronic supplementary material

Below is the link to the electronic supplementary material.

**Supplement table. 1** Comparison of intraoperative conditions.

## Data Availability

The datasets used and/or analyzed during the current study are available from the corresponding author upon reasonable request.

## Abbreviations

Pre-ICNB: Preemptive intercostal nerve block
Post-ICNB: Postoperative ICNB
VATS: Video-assisted thoracic surgery
NRS: Numeric Rating Scales
BCS: Bruggrmann Comfort Scale
ERAS: Enhanced recovery after surgery
TEB: Thoracic epidural blockade
PCA: Patient-controlled analgesia
BIS: Bispectral index
PACU: Post-anesthesia care unit
MMSE: Mini-mental state examination
BMI: Body mass index
ASA level: American Society of Anesthesiologists level
RCRI: Revised cardiac risk index
PVB: Paravertebral block
