# Supplementary material for: Efficacy of preemptive intercostal nerve block on recovery in patients undergoing video-assisted thoracic lobectomy
Source: J Cardiothorac Surg. 2023 Apr 28;18:168. doi: 10.1186/s13019-023-02243-z (PMC10148478; doi:10.1186/s13019-023-02243-z)
Supplement: Supplementary file 1 — Supplement table. 1 Comparison of intraoperative conditions. [file 13019_2023_2243_MOESM1_ESM.doc]

**Supplement table. 1 Comparison of intraoperative conditions.**

| Variable | Pre-ICNB group  (n=23) | Post-ICNB group  (n=25) | *P* value |
| --- | --- | --- | --- |
| Propofol (mg) | 628±212 | 712±275 | 0.131 |
|  |  |  |  |
|  |  |  |  |
| Dexmedetomidine (ug) | 56.29±18.02 | 58.49±29.15 | 0.689 |
| Midazolam (mg) | 2.86±1.04 | 2.84±0.81 | 0.880 |
| Cisatracurium (mg) | 19.95±5.65 | 23.70±11.05 | 0.063 |
|  |  |  |  |
|  |  |  |  |
